# Proteomic profiling of extracellular vesicles derived from human serum for the discovery of biomarkers in Avascular necrosis

**DOI:** 10.1186/s12014-024-09489-2

**Published:** 2024-06-02

**Authors:** Soo-Eun Sung, Ju-Hyeon Lim, Kyung-Ku Kang, Joo-Hee Choi, Sijoon Lee, Minkyoung Sung, Wook-Tae Park, Young-In Kim, Min-Soo Seo, Gun Woo Lee

**Affiliations:** 1 Preclinical Research Center, Daegu-Gyeongbuk Medical Innovation Foundation (K-MEDI hub), Daegu, 41061 Republic of Korea; 2 Korea Biome Research Lab, Kolmar Korea Holdings, 61Heolleungro 8-gil, Seoul, 06800 Republic of Korea; 3 Department of Orthopedic Surgery, Yeungnam University College of Medicine, Yeungnam University Medical Center, 170 Hyonchung-ro, Namgu, Daegu, 42415 Republic of Korea; 4 Cellexobio., Ltd, Daegu, 42415 Korea; 5 Department of Veterinary Tissue Engineering, College of Veterinary Medicine, Kyungpook National University, Daegu, 41566 Republic of Korea

**Keywords:** Avascular necrosis, Osteonecrosis, Extracellular vesicles, Diagnosis

## Abstract

**Background:**

Avascular necrosis (AVN) is a medical condition characterized by the destruction of bone tissue due to a diminished blood supply. When the rate of tissue destruction surpasses the rate of regeneration, effective treatment becomes challenging, leading to escalating pain, arthritis, and bone fragility as the disease advances. A timely diagnosis is imperative to prevent and initiate proactive treatment for osteonecrosis. We explored the potential of differentially expressed proteins in serum-derived extracellular vesicles (EVs) as biomarkers for AVN of the femoral head in humans. We analyzed the genetic material contained in serum-derived exosomes from patients for early diagnosis, treatment, and prognosis of avascular necrosis.

**Methods:**

EVs were isolated from the serum of both patients with AVN and a control group of healthy individuals. Proteomic analyses were conducted to compare the expression patterns of these proteins by proteomic analysis using LC-MS/MS.

**Results:**

Our results show that the levels of IGHV3-23, FN1, VWF, FGB, PRG4, FCGBP, and ZSWIM9 were upregulated in the EVs of patients with AVN compared with those of healthy controls. ELISA results showed that VWF and PRG4 were significantly upregulated in the patients with AVN.

**Conclusions:**

These findings suggest that these EV proteins could serve as promising biomarkers for the early detection and diagnosis of AVN. Early diagnosis is paramount for effective treatment, and the identification of new osteonecrosis biomarkers is essential to facilitate swift diagnosis and proactive intervention. Our study provides novel insights into the identification of AVN-related biomarkers that can enhance clinical management and treatment outcomes.

**Supplementary Information:**

The online version contains supplementary material available at 10.1186/s12014-024-09489-2.

## Background

Avascular necrosis (AVN) is a type of osteonecrosis caused by reduced blood supply to osteocytes and bone marrow [1]. Although it can manifest in various parts of the body, the femoral head, located on the upper part of the femur adjacent to the pelvis, upper arms, shoulders, and knees, is the primary site for this condition [2–5]. Approximately 20,000–30,000 new AVN cases are diagnosed annually in the United States and an estimated 300,000 to 600,000 patients have the condition [6, 7]. The common etiology for AVN can be divided into traumatic and non-traumatic factors. Traumatic factors include hip fractures and dislocations that cause critical ischemia, inflammatory responses, and osteonecrosis. Non-traumatic factors include excessive use of corticosteroids and immunosuppressants, alcohol abuse, Gaucher’s disease, Lupus erythematosus, sickle cell disease, and human immunodeficiency virus (HIV) infection, which all cause osteonecrosis [8–11]. Understanding non-traumatic AVN is challenging for orthopedic surgeons and it is important to elucidate its genetic causes and pathogenic mechanisms [12]. Surgical or nonsurgical treatment can be selected depending on etiological cause, AVN stage, duration of symptoms, pain, age, systemic condition, and whether the disease is unilateral or bilateral [13]. Core decompression (CD) is currently the most common treatment for early stage AVN. The advantage of CD is that it can be performed at an early stage to stop the progression of the disease, or if it fails, other conservative surgeries can be performed [14–16]. However, in order to perform CD, it must be diagnosed at a very early stage to prevent the femoral head collapse from progressing, which is very difficult with current diagnostic methods [17, 18]. The reason for the difficulty of early diagnosis is that many diagnostic methods except magnetic resonance imaging (MRI) have low sensitivity, and the symptoms of early patients are often not clear, so they are often mistaken for spinal diseases [19]. Also, fractures need to be fixed using a metal implant; therefore the use of MRI is limited in patients with bone ischemia after surgery [20]. For these reasons, a rapid diagnosis is essential for the prevention and treatment of osteonecrosis and there is a crucial need to identify biomarkers for diseases with challenging diagnoses, such as cancer.

Recent studies have highlighted the potential use of extracellular vesicles (EVs) as biomarkers for diseases such as cancer. Various studies have shown that EVs harbor specific proteins whose quantities increase in patients with breast cancer, which suggests that they could be potential biomarkers for breast cancer [21, 22]. Extracellular vesicles range from 40 to 200 nm in size, are characterized by phospholipid bilayers, and are ubiquitous in nearly all body fluids, including the cerebrospinal fluid, saliva, breast milk, and blood [23, 24]. The vesicles are released by diverse cell types through the fusion of the plasma membrane with multivesicular bodies and play a crucial role in regulating cell-to-cell communication [25]. They encapsulate proteins, lipids, mRNAs, and miRNAs derived from their originating cells. These constituents remain stable within the EVs, which can traverse the blood-brain barrier [26, 27]. The distinctive characteristics associated with EVs mean that they have been actively investigated as diagnostic tools for various conditions, including cancer, inflammatory bowel disease, and cardiovascular diseases [28–30].

In this study, we analyzed serum-derived EVs to ascertain whether differentially expressed proteins could function as biomarkers for AVN of the femoral head in human patients. The EVs were isolated from the sera of patients with AVN and a control group. Protein expression patterns were then scrutinized using a proteomic analysis. Our findings show that EV proteins could potentially be used as AVN-specific biomarkers.

## Methods

### Serum preparation

All patients diagnosed with AVN at Yeungnam University Hospital between 2021 and 2022 participated in this study. Serum was collected from both the healthy control group (*n* = 11) and the patients with AVN group (*n* = 11) (Table 1). Blood samples were collected in serum-separating tubes (Vacutainer®; Becton Dickinson, Franklin Lakes, NJ, USA) and centrifuged at 1,100 × *g* for 10 min at 4 °C. Supernatants were transferred to 1.7 mL tubes and stored at ˗70 °C until use.

**Table 1:** Participants characterization

Control							AVN							
No.	Age	Gender	No.	Smoking	Alcohol	Underlying disease	No.	Age	Gender	AVN stage	Cause	Smoking	Alcohol	Underlying disease
C-1	35	M	A-1	++	++	-	D-1	63	M	3 A	Alcohol	+++	++	Afib, LC
C-2	51	M	A-2	-	-	HTN	D-2	41	F	2 C	Idiopathic	-	-	Hypothyroidisim
C-3	50	M	A-3	++	++	-	D-3	55	M	4	Idiopathic	++	++	-
C-4	40	M	A-4	-	++	-	D-4	64	M	4	Idiopathic	-	-	Osteoprosis
C-5	36	M	A-5	++	+	-	D-5	48	M	4	Idiopathic	++	++	-
C-6	31	M	A-6	+	+	-	D-6	54	M	3B	Alcohol	+++	++	LC
C-7	28	M	A-7	-	-	-	D-7	70	F	4	RA	-	-	RA
C-8	26	M	A-8	+	+	-	D-8	80	M	4	Alcohol	+++	++	Hypothyroidism, Tb, Afib, HBV
C-9	29	M	A-9	-	-	-	D-9	41	M	3B	Idiopathic	-	-	-
C-10	27	M	A-10	+	-	-	D-10	47	M	3B	Idiopathic	++	-	-
C-11	52	M	A-11	+	-	HTN	D-11	80	M	4	Idiopathic	++	-	HTN, CKD

Drinking history was marked with a ‘+’ if the person drank 1 bottle every day for 10 years

Smoking history is indicated by a ‘+’ if the person smoked 1 pack a day for 10 years

The AVN stage was classified using the Association for Research on Circulating Fractures classification (ARCO).

Afib : Atrial fibrillation ; LC : Liver cirrhosis ; RA : Rheumatoid arthritis ; Tb : Tuberculosis ; HBV : hepatitis B virus ; HTN : hypertension ; CKD : chronic kidney disease

### EV isolation and characterization

EVs were isolated from human serum using ExoQuick® (System Biosciences, Palo Alto, CA, USA), following the manufacturer’s protocol. The isolated EVs were analyzed for total protein levels using the bicinchoninic acid (BCA) assay (Thermo Fisher Scientific, Waltham, MA, USA). To determine the size and concentration of EVs, a nanoparticle tracking analyzer (NS100, Malvern Panalytical, UK) was employed. The EV surface markers CD63 (BioLegend, San Diego, CA, USA) and CD81 (BioLegend) were analyzed using flow cytometry (NovoCyte™; Agilent, Santa Clara, CA, USA), and the data were visualized using NovoExpress® 1.5.6 software (Agilent Technologies). For EV morphology imaging, samples were placed on a Formvar® carbon 200 mesh (Ted Pella, Redding, CA, USA), fixed with 4% paraformaldehyde for 10 min, and observed using a bio-TEM (HT7700, Hitachi, Japan) instrument.

### FASP digestion

FASP digestion was employed for protein quantification using the BCA assay following the manufacturer’s protocol (Pierce BCA protein Assay Kit, Thermo Fisher). For each sample, 100 µg of protein was used. Tris(2-carboxyethyl)phosphine (final concentration 5 mM) was added, and the samples were incubated with shaking at 300 rpm for 30 min at 37 °C for protein reduction. Subsequently, the samples were centrifuged at 14,000 × *g* for 15 min. Indol-3-acetic acid (final concentration, 50 mM) was added for protein alkylation, and the samples were incubated for 1 h in the dark, followed by centrifugation at 14,000 × *g* for 15 min. Thereafter, 100 µL of 8 M urea (in 0.1 M Tris/HCl, pH 8.5) was added, and the samples were centrifuged at 14,000 × *g* for 15 min, repeating the process thrice. Next, 100 µL of 50 mM ammonium bicarbonate was added and the solution was centrifuged at 14,000 × *g* for 15 min. Trypsin, dissolved in 50 mM ammonium bicarbonate, was used for peptide digestion while shaking at 300 rpm for 18 h at 37 °C. Subsequently, 40 µL of 50 mM ammonium bicarbonate were added, and all solutions were vacuum filtered. The protein digestion was terminated by adding 15 µL of formic acid (pH 2).

### Desalting

Desalting was performed using a C18 Microspin column with 50 µL of 0.1% formic acid and 80% acetonitrile (in 0.1% formic acid) in 100 µL. The samples were then dried with a speed-vac to remove all solutions before being stored at ˗20 °C before analysis.

### Liquid chromatography–mass spectrometry (LC–MS) data acquisition

Protein analysis was conducted using a Dionex UltiMate™ 3000 system (Dionex, Sunnyvale, CA, USA) and Thermo Q-Exactive™ MS (Thermo Scientific, San Jose, CA, USA). Purification of samples was performed using a trapping column (C_18_, 3 μm, 100 Å, 75 μm × 2 cm). The purified samples were separated using an analytical column (PepMap™ RSLC C_18_, 2 μm, 100 Å, 75 μm × 50 cm). The sample flow rate was set at 300 nL/min with the following gradient: 0–14 min sustaining 4% solvent B, 14–120 min ramping solvent B to 40%, 120–130 min ramping solvent B to 96%, and 130–180 min decreasing solvent B to 4%. Solvent A comprised 0.1% formic acid in water, whereas solvent B consisted of 80% acetonitrile and 0.1% formic acid. The separation time was 180 min, and the mass range was set at 400–8,000 m/z. Proteome Discoverer™ software 2.5 (Thermo Scientific) was utilized for data analysis and the human database was downloaded from Uniport.

### Proteome data analysis

The appropriate processing workflow included Spectrum Files RC for calculation and recalibrating precursor masses and SEQUEST HT process to detect as a database search algorithm. Precursor abundance calculation was based on intensity. The search parameters were set up as follows: 10 ppm of tolerances of precursor ion masses, 0.02 Da fragment ion mass, and a maximum of two missed cleavages with trypsin enzyme. After searching, the data results below 1% of FDR were selected and filtered at least six more peptide lengths. Sample normalization utilized the Proteome Discoverer™ software 2.5 total peptide amount method [31]. Fold change was calculated in the protein abundance-based ratio. *P*-values were calculated for the reported quantification ratios using the ANOVA test based on individual proteins. Differentially expressed proteins were selected based on p-value < 0.05, |log2 fold change| > 1. Proteins with *p*-value < 0.05 and fold change > 2.0 were regarded as up-regulated, while proteins with *p*-value < 0.05 and fold change < 1/2 were regarded as down-regulated.

### Enzyme-linked immunosorbent assay (ELISA)

The EV samples were scrutinized for several upregulated markers. ELISA was performed to detect von Willebrand factor (VWF; MyBioSource, MBS704140, Canada) and PRG4 (MyBioSource, MBS700233), following the manufacturer’s instructions. All tests were conducted in triplicate per sample.

### Statistical analysis

The results were analyzed using GraphPad Prism software (San Diego, CA, USA). Statistical analyses were performed using a two-tailed Student’s *t*-test, and all data are presented as means ± SD.

## Results

### Isolation of EVs

**Fig. 1:**
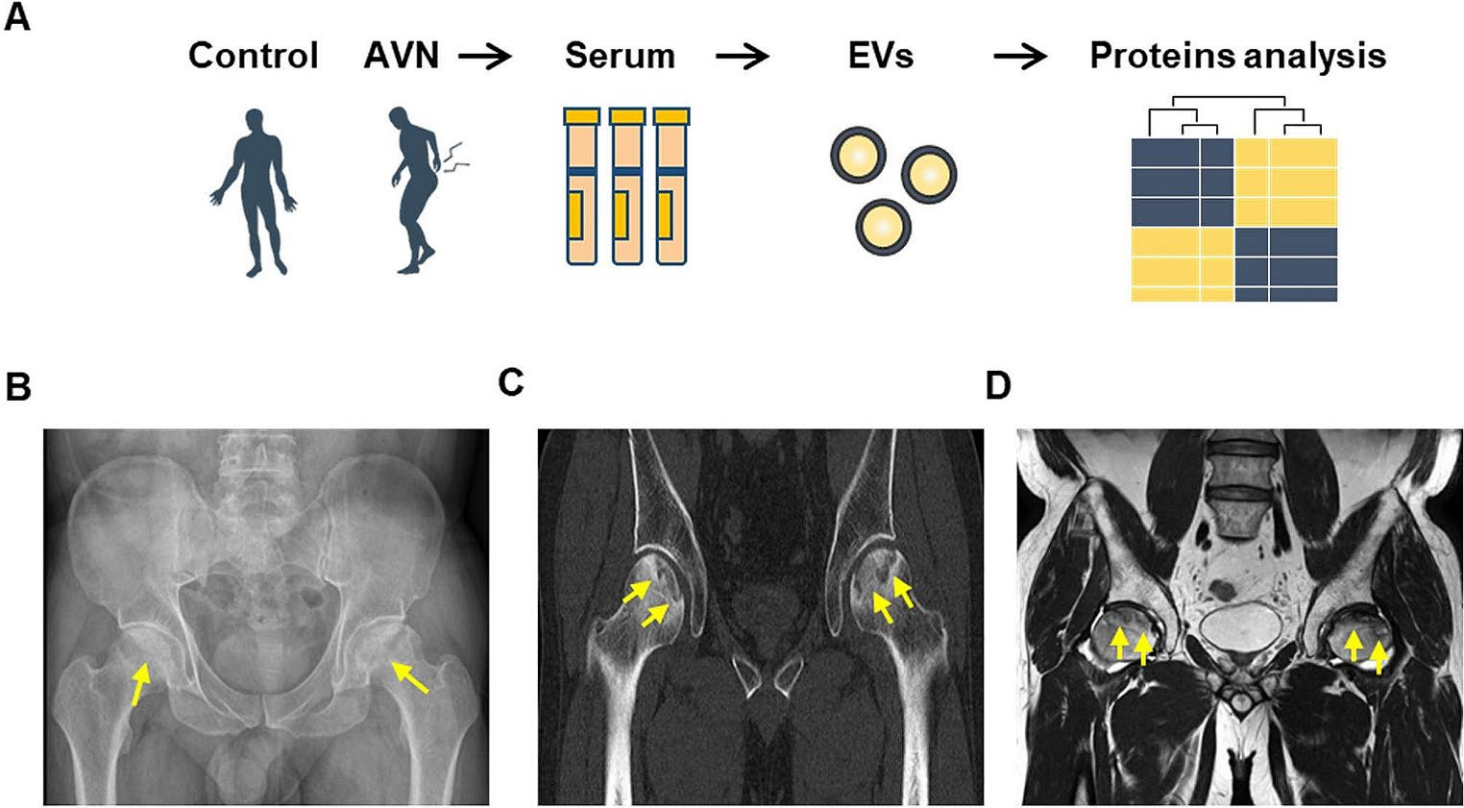
Isolation of human serum-derived EVs and quantification of isolated EVs. (**A**) Study design comparing protein expression patterns in human serum-derived EVs from a control and AVN groups. (**B**) Simple radiograph displaying bilateral femoral head collapse due to AVN (yellow arrows). (**C**) Computed tomography (CT) scan depicting bilateral femoral head collapse with AVN (yellow arrows). (**D**) MR T2-weighted image showing bilateral femoral head collapse with low signal intensity in the femoral head (yellow arrows)

Our study aimed to validate the protein expression patterns of human serum-derived EVs in the control group and patients with AVN (Fig. 1A). The phenotypes of the samples and individual sample information are outlined in Table 1. The average age of the control individuals was 36.8 years, and all patients were male. The average age of the patients with AVN was 58.5 years, with 81.8% being male. The results of AVN diagnosis through CT and MR are presented in Fig. 1B–D.

### Characterization of EVs

**Fig. 2:**
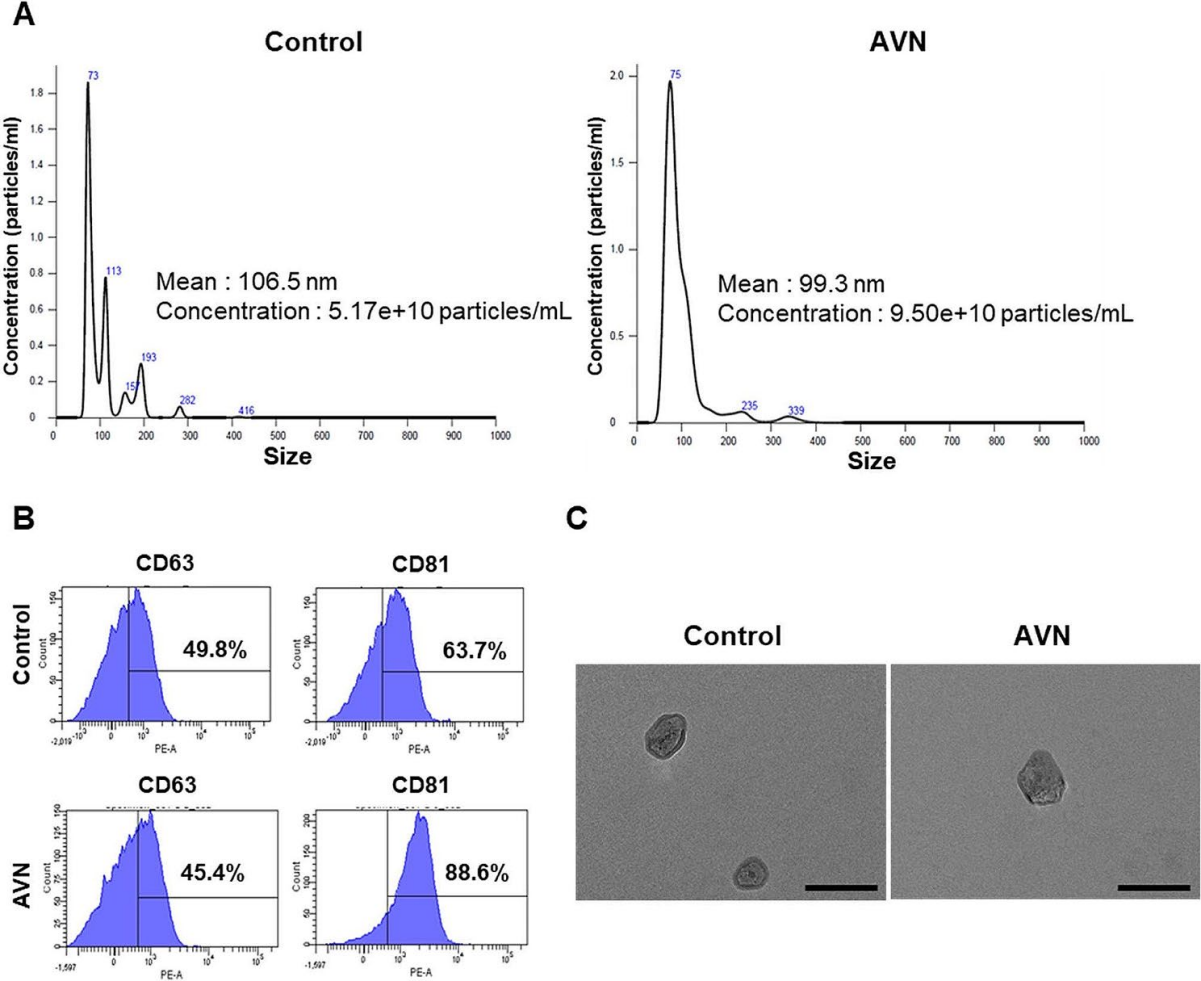
Characterization of serum-derived EVs. (**A**) NTA of EVs to confirm particle nature and size distribution. (**B**) Flow cytometry assay of EV markers CD63 and CD81. (**C**) TEM image displaying EV morphology and size. Scale bar = 200 nm

Human serum-derived EVs were characterized prior to the analysis of their protein expression patterns. Further, nanoparticle tracking analysis (NTA) was conducted to confirm the size and particle concentration. The size of EVs ranged from 40 to 150 nm (Fig. 2A). Isolated EVs were detected in both the control (5.17 × 10^10^ particles/mL) and patients with AVN groups (9.50 × 10^10^ particles/mL). Flow cytometry confirmed the presence of tetraspanins, such as CD63 and CD81, revealing that 49.8% of EVs from the control group were CD63-positive and 63.7% were CD81-positive. In the AVN group, flow cytometry showed that EVs comprised 45.4% CD63-positive and 88.6% CD81-positive cells (Fig. 2B). Lastly, transmission electron microscope images illustrated that the EVs isolated from the serum exhibited bilayer membranes, a spherical shape, and an approximate diameter of 100 nm (Fig. 2C). Collectively, these results affirmed that the nanoparticles isolated from human serum were EVs.

### LC–MS analysis of EV proteins in the control and AVN groups

**Fig. 3:**
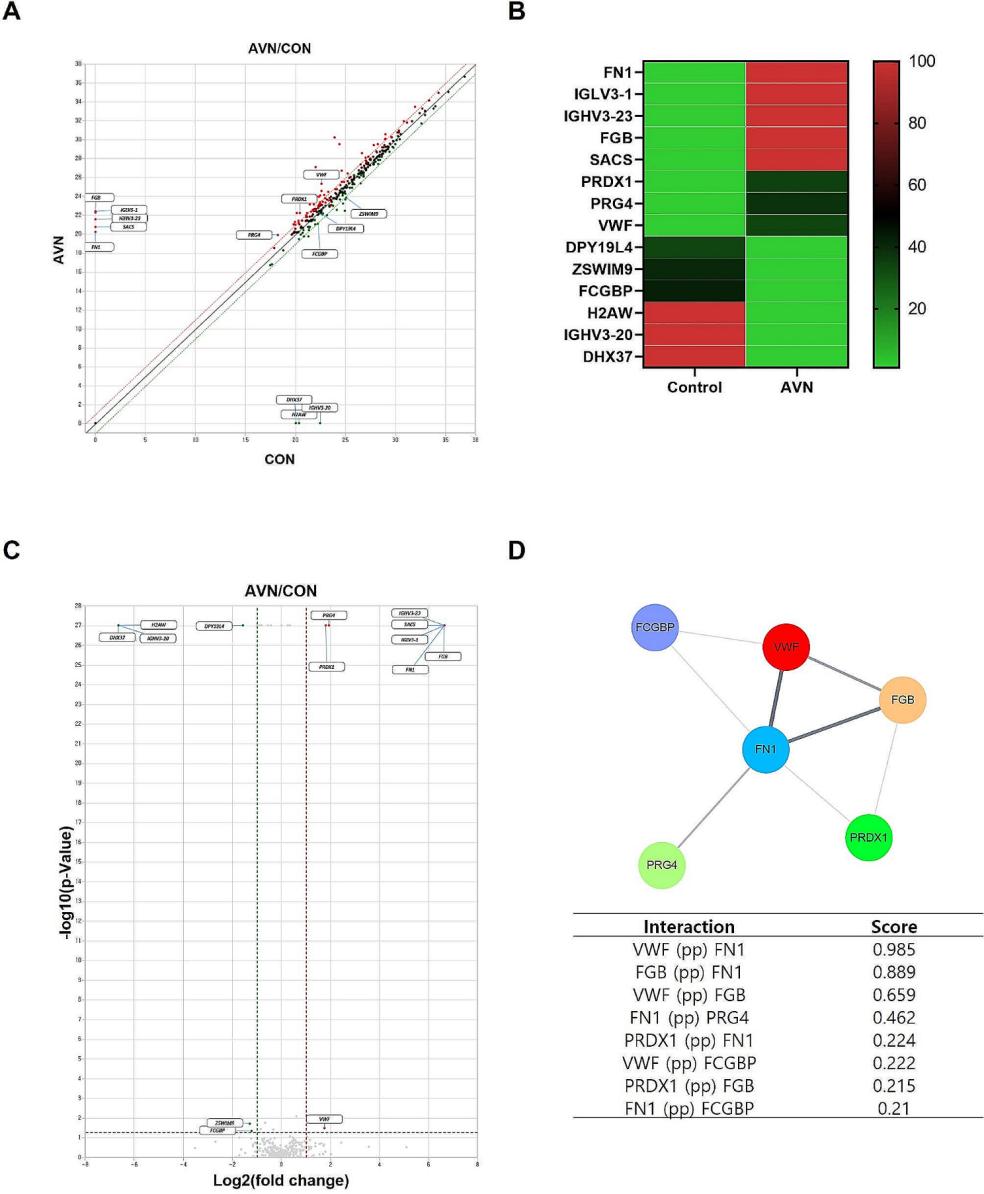
Proteomic analysis of EV proteins. (**A**) Scatter plot presenting upregulated and downregulated proteins. Red spots indicate an increase in patients with AVN, and green spots show a decrease in patients with AVN. (**B**) Heatmap of the expression pattern, indicating proteins with greater than two-fold differential expression (scale bar = relative value). (**C**) Volcano plot illustrating the distribution of differentially expressed proteins. (**D**) Network of protein interactions, with thick lines indicating strong interactions between proteins

The EV proteins were analyzed using LC–MS to identify AVN-specific EV protein markers (Table S1). Scatter plots and heatmaps were generated to discern specific EV protein expression patterns in each group. Figure 3A displays a scatter plot illustrating protein expression levels between the control and AVN groups, highlighting several proteins with more than a two-fold difference. The heatmap in Fig. 3B represents the relative expression levels of 14 proteins that differed by more than a two-fold change, displayed as a colored bar.

In the analysis of EV proteins, fibronectin 1 (FN1), VWF, immunoglobulin heavy variable (IGHV)3–23, fibrinogen beta chain (FGB), immunoglobulin lambda variable 3 − 1 (IGLV3-1), proteoglycan 4 (PRG4), peroxiredoxin-1 (PRDX1), and spastic ataxia of Charlevoix (SACS) levels were elevated in patients with AVN. In contrast, levels of histone H2A type 3 (H2AW), IGHV3-20, Fc gamma-binding protein (FCGBP), DEAH-box helicase 37, dpy-19-like 4 (DPY19L4), and zinc finger SWIM-type containing 9 (ZSWIM9) were lower in patients with AVN than in controls (Table 2). The significance was confirmed using volcano plots (Fig. 3C). Patients with AVN exhibited higher levels of VWF, FN1, PRG4, and PRDX1, whereas the levels of H2AW, DPY19L4, ZSWIM9, and FCGBP were decreased in the AVN group.

To explore protein–protein interactions, Cytoscape software was employed to analyze 14 proteins and generate a network map illustrating interactions among these proteins, chosen based on their known roles in the signaling pathways of interest (Fig. 3D). The six proteins showed interactions with each other, with FN1 and VWF displaying the closest interactions. Notably, the closer the score to 1, the more closely related it was. These findings suggest that the altered proteins in the AVN group have the potential to serve as AVN biomarkers.

**Table 2:** Significant differentially expressed proteins

No.	Proteins	Fold change	Average of normalized data (log2)		Up/down	Normalized data (log2)					
			AVN	CON		AVN1	AVN2	AVN3	CON1	CON2	CON3
1	FN1	100	20.25	.	UP	21.04	.	18.42	.	.	.
2	VWF	3.379	25.35	22.61	UP	26.29	24.41	24.48	23.03	22.73	21.79
3	IGHV3-23	100	21.57	.	UP	22.44	.	19.02	.	.	.
4	FGB	100	22.26	.	UP	22.65	.	21.73	.	.	.
5	IGLV3-1	100	22.41	.	UP	22.41	.	.	.	.	.
6	PRG4	3.827	19.91	18.23	UP	20.17	20.45	18.43	18.23	.	.
7	PRDX1	3.493	22.25	20.45	UP	22.25	.	.	20.45	.	.
8	SACS	100	20.77	.	UP	20.77	.	.	.	.	.
9	H2AW	0.01	.	20.36	Down	.	.	.	20.36	.	.
10	IGHV3-20	0.01	.	22.49	Down	.	.	.	.	22.49	.
11	FCGBP	0.43	21.11	22.26	Down	20.61	21.43	21.18	21.62	22.39	22.59
12	DHX37	0.01	.	20.04	Down	.	.	.	20.04	.	.
13	DPY19L4	0.339	22.30	22.34	Down	.	23.25	18.31	.	22.34	.
14	ZSWIM9	0.409	23.86	25.08	Down	24.24	23.49	23.76	25.05	24.93	25.24

FN1 (fibronectin 1); VWF (von Willebrand factor); IGHV3-23 (immunoglobulin heavy variable 3–23); FGB (fibrinogen beta chain); IGLV3-1 (Immunoglobulin lambda variable 3 − 1); PRG4 (proteoglycan 4); PRDX1 (peroxiredoxin-1); SACS (spastic ataxia of Charlevoix-Saguenay); H2AW (Histone H2A type 3); IGHV3-20 (immunoglobulin heavy variable 3–20); FCGBP (Fc Gamma Binding Protein); DHX37 (DEAH-box helicase 37); DPY19L4 (dpy-19 like 4); ZSWIM9 (zinc finger SWIM-type containing 9)

### Validation of EV protein in the control and AVN groups

**Fig. 4:**
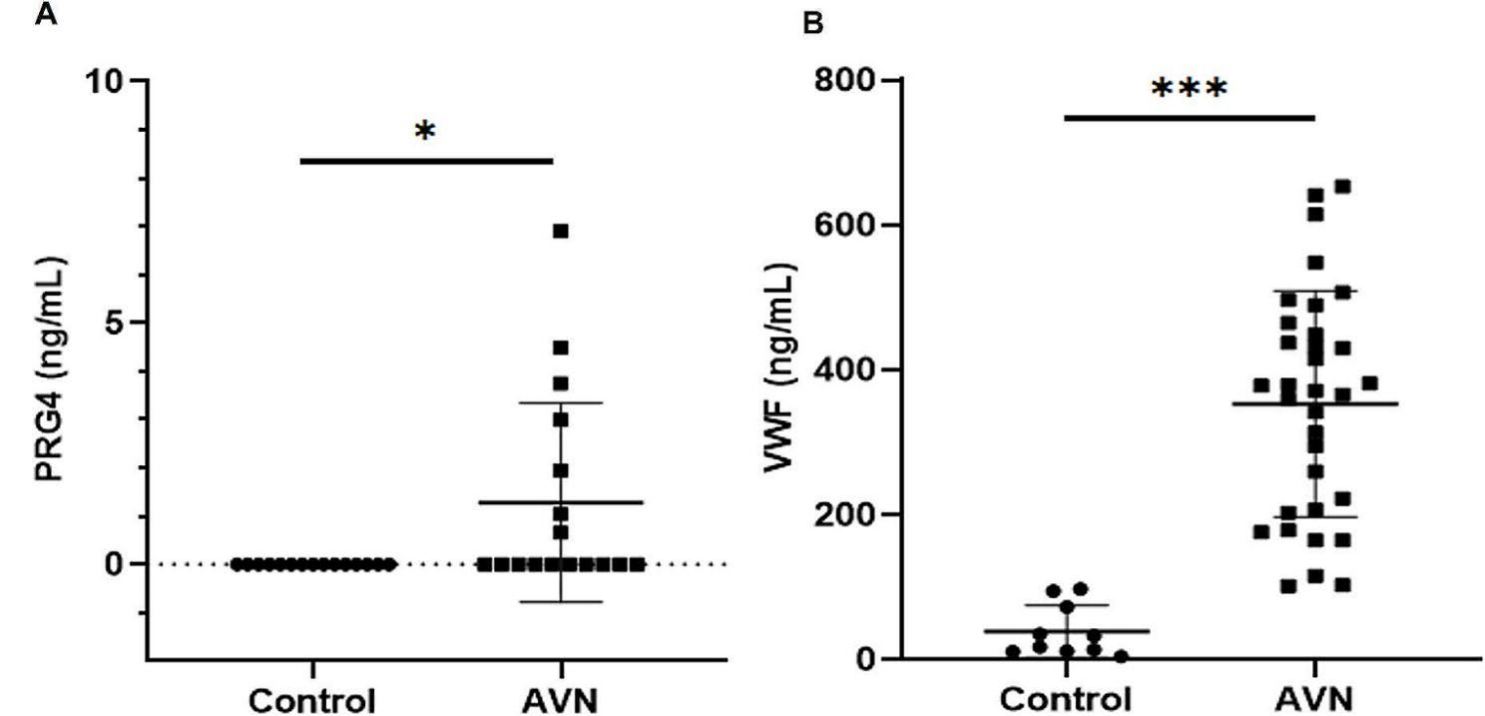
EV protein analysis using ELISA. (**A**, **B**) ELISA data depicting the expression of five EV proteins, comparing the AVN group with the control group. VWF (von Willebrand factor) and PRG4 (proteoglycan 4). The data are expressed as means ± SD; *p < 0.05, ***p < 0.001

Among the upregulated proteins, IGLV3-1, PRDX1, and SACS exhibited significant expression in patients with AVN (Table 2). ELISA was conducted for VWF and PRG4, both of which were upregulated in all patients with AVN. The expression levels of VWF and PRG4 were significantly higher in the AVN group than in the control group (Fig. 4A, B). EV protein analysis revealed that the VWF concentrations were 38.8 and 352.9 ng/mL in the control and AVN groups, respectively. PRG4 was not detected in the control group but averaged 1.283 ng/mL in the AVN group. ELISA confirmed that VWF and PRG4 levels were significantly increased in the AVN group.

## Discussion

This study aimed to identify and analyze specific proteins that could potentially serve as biomarkers associated with patients with AVN. EVs were isolated from the sera of healthy controls and patients with AVN, and the expression patterns of EV proteins were analyzed. The use of EV composition as a disease-specific biomarker for various diseases has been suggested because EV components reflect origin cell properties [17, 18]. In particular, EV proteins may be candidates for personalized cancer treatment and prognosis in cancer [32]. Recently, EV PD-L1 protein has been revealed to be targeted by anti-PD-L1 therapeutic immunosuppression in tumors [33, 34]. EV PD-L1 as a blood-based biomarker is an ideal strategy compared to an invasive tumor biopsy. Our study aimed to identify biomarkers using EV proteins for osteonecrosis, an orthopedic disease, and present them as potential diagnostic markers to help diagnose AVN. In this study, serum-derived EVs were isolated and characterized. The isolated particles were confirmed to be EVs using TEM, NTA, and flow cytometry.

MicroRNAs and EV proteins have been scrutinized using bioinformatics methods [35, 36]. In this study, our focus was on identifying potential AVN diagnostic biomarkers by analyzing the expression of specific proteins in EVs derived from the sera of patients with AVN compared to those derived from normal controls. The results, based on LC–MS analysis, indicated that FN1, VWF, IGHV3-23, FGB, IGLV3-1, PRG4, PRDX1, and SACS were highly expressed in the EVs derived from the serum of patients with AVN compared with the serum EVs of the control group.

FN1 is involved in cell adhesion and migration during embryogenesis, wound healing, blood coagulation, host defense, and metastasis [37, 38]. It is also a high-molecular-weight glycoprotein present in connective tissue, blood, and on the surface of cells. Notably, FN1 is a potential biomarker for the diagnosis of ovarian cancer [39], bladder cancer [40], and glioblastoma [41]. However, the correlation between FN1 and AVN has not yet been clearly established. VWF, a blood multimeric protein with a very high molecular weight, is involved in primary hemostasis and the physiological process of platelet attachment to damaged blood vessel walls. Mutations in VWF cause von Willebrand disease, a hereditary bleeding disorder [42, 43]. VWF plays a role in various physiological processes, including vascular permeability, inflammation, and angiogenesis. The absence of VWF, as observed in type 3 von Willebrand disease, is associated with increased vascularization and severe clinical manifestations, such as gastrointestinal bleeding due to vascular malformations [44]. Studies on biomarkers related to VWF and osteonecrosis of the femoral head have reported upregulation of VWF expression in patients with osteonecrosis [45, 46]. However, research on EV proteins in VWF and AVN diseases is yet to be reported.

IGHV3 is the most commonly used subgroup in B-cell chronic lymphocytic leukemia (CLL), followed by IGHV1 and IGHV4. Two genes within the IGHV3 subgroup—IGHV3-23 and IGHV3-7—are among the most frequently investigated IGHV genes in this leukemia, alongside IGHV1-69 and IGHV4-34. The IGHV3 genes, particularly IGHV3-23 and IGHV3-7, exhibit some of the highest mutational loads in CLL [47]. However, a clear correlation between IGHV3 and AVN has not been reported.

FGB is a thrombin-clotting glycoprotein found in mammalian blood. The primary structures of human fibrinogen alpha, beta, and gamma polypeptide chains have been identified through amino acid and nucleic acid sequencing. The primary physiological function of fibrinogen is to form fibrin, which binds to platelets and plasma proteins in hemostatic plugs. Fibrinogen plays a crucial role in platelet aggregation [48, 49]. Although FGB has been investigated as a potential biomarker for various diseases, including preeclampsia, gastric carcinoma, disseminated intravascular coagulation, and oral squamous cell carcinoma [50–53], the relationship between FGB and AVN remains unclear. IGLV3-1 is upregulated in the serum of patients with coronavirus disease 2019 and has been suggested as a biomarker [54], but the correlation between IGLV3-1 and AVN is yet to be reported.

PRDX1 belongs to a family of thiol-specific antioxidant proteins influencing hydrogen peroxide levels, mediating signal transduction pathways [55], and playing roles in cell proliferation, differentiation, and apoptosis [56, 57]. PRG4 is a macromolecule found on the chondrocyte surface with diverse biological functions, including immunoreactivity, cytoprotection, lubrication, and matrix binding [58, 59]. It is expressed in various tissues, such as the lungs, heart, liver, bone, and cartilage, and can be detected in the serum and synovial fluid [60]. Although PRG4 has been studied as a biomarker for diseases like chronic obstructive pulmonary disease [61], sepsis [62], osteoarthritis [63], and hepatocellular carcinoma [64], correlation between PRG4 levels and AVN has not been reported.

PRDX1, has been identified as a biomarker helpful in the diagnosis of several diseases, including ovarian cancer [55], abdominal aortic aneurysm [65], pancreatic cancer [66], and irritable bowel syndrome [67]. Nevertheless, the potential use of PRDX1 as a biomarker for AVN remains to be studied.

Autosomal recessive SACS–Saguenay (ARSACS) is a rare early-onset neurodegenerative disease caused by mutations in the SACS gene, encoding sacsin [68]. The ARSACS case discovered in Quebec presented with a phenotype characterized by cerebellar ataxia, spasticity, and polyneuropathy [69]. However, the correlation between SACS and AVN has not yet been reported.

As demonstrated, eight upregulated proteins were confirmed in AVN group using LC–MS. Among them, VWF and PRG4, proteins expressed in all patients with AVN, were verified using ELISA, confirming the same elevated expression patterns observed using LC–MS. Therefore, VWF and PRG4 could be potential novel biomarkers for diagnosing AVN with EV proteins. In summary, EVs were successfully isolated from human serum, and the protein expression patterns of EVs from patients with AVN and controls were compared and analyzed to identify changes in the expression of specific proteins. Consequently, we identified specific protein biomarkers potentially associated with AVN. A rapid and accurate diagnosis of avascular osteonecrosis may be achievable through a diagnostic method based on biochemical markers, rather than relying solely on existing diagnostic methods, such as radiography and CT.

To further our understanding of the identified proteins in the context of AVN, we compared our findings with existing metabolic and transcriptomic studies [70, 71]. The metabolic pathways, particularly influenced by systemic conditions such as steroid use, offer a broader insight into the pathophysiology of ONFH. Proteins like VWF, although primarily involved in hemostasis, may also reflect endothelial dysfunctions exacerbated by metabolic disturbances. Similarly, FN1, through its role in tissue integrity and cellular adhesion, could indicate metabolic stress within bone tissues, which is crucial for understanding the extracellular matrix alterations in ONFH. Transcriptomic analyses complement our proteomic data, highlighting upregulations in genes associated with immune responses and inflammation, similar to the profiles of IGHV3-23 and PRG4 observed in our study. These findings suggest that the proteins identified may serve as biomarkers for AVN and reflect broader metabolic and genetic disturbances affecting the disease process. This integrative approach enhances the potential diagnostic value of our findings, proposing these proteins as multifaceted biomarkers that reflect both the localized bone pathology and systemic disease mechanisms.

Recent advances in metabolomics, particularly those related to the metabolic profiling of bone-derived exosomes in ONFH, have brought significant insights into the pathophysiology of avascular necrosis. Key pathways such as riboflavin metabolism, pantothenate and CoA biosynthesis, glycerophospholipid metabolism, and sphingolipid metabolism have been highlighted as significantly altered in ONFH patients [72]. Intricately linked with cellular energy metabolism and lipid homeostasis, these pathways play a crucial role in maintaining bone tissue integrity under ischemic conditions. Integrating these metabolomic findings with our proteomic data provides a more comprehensive molecular picture of ONFH, suggesting that disruptions in these metabolic pathways could contribute to the disease’s progression by impairing bone cell viability and function.

Our study had some limitations. To our knowledge, this is the first study to identify EV biomarkers of AVN in the femoral head. Therefore, we enrolled only patients definitively diagnosed with AVN and scheduled them for arthroplasty. In other words, all enrolled patients were at the end-stage of the disease, as we aimed to differentiate between those with and without the disease. Subsequently, we plan to conduct future research on changes in EV biomarkers according to AVN disease stage. Further, our study sample consisted of only 11 patients, and a larger sample size should be analyzed in the future. Subsequently, in future studies, we plan to collect samples from > 100 patients with AVN to confirm the changes in the EV biomarkers presented in this study. Nevertheless, this study is the first to evaluate the feasibility of developing EV biomarkers for orthopedic diseases and could be regarded as a pilot trial for such a biomarker study. Further studies are necessary to confirm the accuracy of the identified biomarkers and their applicability in clinical practice.

## Conclusions

Osteonecrosis is a debilitating condition that leads to pain, arthritis, and bone fragility. Early diagnosis is crucial for effective treatment, and the identification of new biomarkers of osteonecrosis is necessary to facilitate rapid diagnosis and proactive treatment. Our results show that the levels of VWF and PRG4 were upregulated in the EVs of patients with AVN compared with those of healthy controls. These findings suggest that EV proteins could serve as potential biomarkers for early detection and diagnosis of AVN. Based on these results, we hope to collect more patient samples and prove it through additional verification in order to apply it to clinical stages.

### Electronic supplementary material

Below is the link to the electronic supplementary material.

Supplementary Material 1

## Data Availability

Complete datasets for the proteomics LC?MS results are included in the Supplementary table.xls file.

## Abbreviations

ARSACS: Autosomal recessive spastic ataxia of Charlevoix?Saguenay
AVN: Avascular necrosis
BCA: Bicinchoninic acid
CT: Computed tomography
DPY19L4: Dpy-19-like 4
ELISA: Enzyme-linked immunosorbent assay
EVs: Extracellular vesicles
FCGBP: Fc gamma-binding protein
FGB: Fibrinogen beta chain
FN1: Fibronectin 1
H2AW: Histone H2A type 3
IGHV: Immunoglobulin heavy variable
IGLV3-1: Immunoglobulin lambda variable 3 − 1
LC–MS: Liquid chromatography–mass spectrometry
NTA: Nanoparticle tracking analysis
ONFH: Osteonecrosis of the femoral head
PRDX1: Peroxiredoxin-1
PRG4: Proteoglycan 4
VWF: Von Willebrand factor
ZSWIM9: Zinc finger SWIM-type containing 9

## References

[1] Wells ME, Dunn JC (2022). Pathophysiology of avascular necrosis. Hand Clin.

[2] Afshar A, Tabrizi A (2020). Avascular necrosis of the Carpal bones Other Than Kienbock Disease. J Hand Surg Am.

[3] Betz C, Mehling IM, Sauerbier M (2015). [Bone necrosis of the Hand]. Z Orthop Unfall.

[4] Migliorati CA, Brennan MT, Peterson DE. Medication-related osteonecrosis of the Jaws. J Natl Cancer Inst Monogr, 2019(53). 10.1093/jncimonographs/lgz009 31425596

[5] Wax A, Leland R (2019). Freiberg Disease and Avascular necrosis of the metatarsal heads. Foot Ankle Clin.

[6] Moya-Angeler J, Gianakos AL, Villa JC, Ni A, Lane JM (2015). Current concepts on osteonecrosis of the femoral head. World J Orthop.

[7] Aldridge JM, Urbaniak JR (2004). Avascular necrosis of the femoral head: etiology, pathophysiology, classification, and current treatment guidelines. Am J Orthop (Belle Mead NJ).

[8] Chang C, Greenspan A, Gershwin ME (2020). The pathogenesis, diagnosis and clinical manifestations of steroid-induced osteonecrosis. J Autoimmun.

[9] Wang A, Ren M, Wang J (2018). The pathogenesis of steroid-induced osteonecrosis of the femoral head: a systematic review of the literature. Gene.

[10] Huang C, Wen Z, Niu J, Lin S, Wang W (2021). Steroid-Induced Osteonecrosis of the femoral head: Novel Insight into the roles of Bone endothelial cells in Pathogenesis and Treatment. Front Cell Dev Biol.

[11] Yombi JC, Vandercam B, Wilmes D, Dubuc JE, Vincent A, Docquier PL (2009). Osteonecrosis of the femoral head in patients with type 1 human immunodeficiency virus infection: clinical analysis and review. Clin Rheumatol.

[12] Pouya F, Kerachian MA (2015). Avascular necrosis of the femoral head: are any genes involved?. Arch Bone Jt Surg.

[13] Ulusoy I, Yilmaz M, Kivrak A (2023). Efficacy of autologous stem cell therapy in femoral head avascular necrosis: a comparative study. J Orthop Surg Res.

[14] Tripathy SK, Goyal T, Sen RK (2015). Management of femoral head osteonecrosis: current concepts. Indian J Orthop.

[15] Soohoo NF, Vyas S, Manunga J, Sharifi H, Kominski G, Lieberman JR (2006). Cost-effectiveness analysis of core decompression. J Arthroplasty.

[16] Andronic O, Weiss O, Shoman H, Kriechling P, Khanduja V (2021). What are the outcomes of core decompression without augmentation in patients with nontraumatic osteonecrosis of the femoral head?. Int Orthop.

[17] Dermawan JK, Goldblum A, Reith JD, Kilpatrick SE (2021). Accurate and Reliable diagnosis of avascular necrosis of the femoral head from total hip arthroplasty specimens requires pathologic examination. Am J Clin Pathol.

[18] Konarski W, Pobozy T, Sliwczynski A, Kotela I, Krakowiak J, Hordowicz M, Kotela A. Avascular necrosis of femoral head-overview and current state of the art. Int J Environ Res Public Health 2022. 19(12). 10.3390/ijerph19127348 PMC9223442 35742595

[19] Li WL, Tan B, Jia ZX, Dong B, Huang ZQ, Zhu RZ, Zhao W, Gao HH, Wang RT, Chen WH (2020). Exploring the risk factors for the misdiagnosis of osteonecrosis of femoral head: a case-control study. Orthop Surg.

[20] Large TM, Adams MR, Loeffler BJ, Gardner MJ (2019). Posttraumatic avascular necrosis after proximal femur, proximal Humerus, Talar Neck, and scaphoid fractures. J Am Acad Orthop Surg.

[21] Joyce DP, Kerin MJ, Dwyer RM (2016). Exosome-encapsulated microRNAs as circulating biomarkers for breast cancer. Int J Cancer.

[22] Wang M, Ji S, Shao G, Zhang J, Zhao K, Wang Z, Wu A (2018). Effect of exosome biomarkers for diagnosis and prognosis of breast cancer patients. Clin Transl Oncol.

[23] Doyle LM, Wang MZ. Overview of Extracellular vesicles, their origin, composition, purpose, and methods for Exosome isolation and analysis. Cells 2019, 8(7). 10.3390/cells8070727 PMC6678302 31311206

[24] Sung SE, Kang KK, Choi JH, Lee SJ, Kim K, Lim JH, Yang SY, Kim SK, Seo MS, Lee GW. Comparisons of Extracellular vesicles from Human Epidural Fat-derived mesenchymal stem cells and fibroblast cells. Int J Mol Sci. 2021; 22(6). 10.3390/ijms22062889 PMC8000612 33809214

[25] Shah R, Patel T, Freedman JE (2018). Circulating Extracellular vesicles in Human Disease. N Engl J Med.

[26] Lu M, Xing H, Xun Z, Yang T, Ding P, Cai C, Wang D, Zhao X (2018). Exosome-based small RNA delivery: Progress and prospects. Asian J Pharm Sci.

[27] Saint-Pol J, Gosselet F, Duban-Deweer S, Pottiez G, Karamanos Y. Targeting and crossing the blood-brain barrier with Extracellular vesicles. Cells 2020; 9(4). 10.3390/cells9040851 PMC7226770 32244730

[28] Abdul Rehman S, Khurshid Z, Hussain Niazi F, Naseem M, Al Waddani H, Sahibzada HA. Sannam Khan R: role of salivary biomarkers in detection of Cardiovascular diseases (CVD). Proteomes 2017; 5(3). 10.3390/proteomes5030021 PMC5620538 28783097

[29] Henning RJ (2021). Cardiovascular exosomes and MicroRNAs in Cardiovascular Physiology and Pathophysiology. J Cardiovasc Transl Res.

[30] Zheng X, Chen F, Zhang Q, Liu Y, You P, Sun S, Lin J, Chen N (2017). Salivary exosomal PSMA7: a promising biomarker of inflammatory bowel disease. Protein Cell.

[31] Weiner S, Sauer M, Visser PJ, Tijms BM, Vorontsov E, Blennow K, Zetterberg H, Gobom J (2022). Optimized sample preparation and data analysis for TMT proteomic analysis of cerebrospinal fluid applied to the identification of Alzheimer’s disease biomarkers. Clin Proteom.

[32] Wang X, Tian L, Lu J, Ng IO (2022). Exosomes and cancer - diagnostic and prognostic biomarkers and therapeutic vehicle. Oncogenesis.

[33] Xie F, Xu M, Lu J, Mao L, Wang S (2019). The role of exosomal PD-L1 in tumor progression and immunotherapy. Mol Cancer.

[34] Chen G, Huang AC, Zhang W, Zhang G, Wu M, Xu W, Yu Z, Yang J, Wang B, Sun H (2018). Exosomal PD-L1 contributes to immunosuppression and is associated with anti-PD-1 response. Nature.

[35] Sung M, Sung SE, Kang KK, Choi JH, Lee S, Kim K, Lim JH, Lee GW, Rim HD, Kim BS et al. Serum-Derived Neuronal Exosomal miRNAs as Biomarkers of Acute Severe Stress. Int J Mol Sci. 2021, 22(18). 10.3390/ijms22189960 PMC8470330 34576126

[36] Sung M, Sung SE, Kang KK, Choi JH, Lee S, Kim K, Lim JH, Lee GW, Rim HD, Won S et al. Serum-derived neuronal exosomal microRNAs as stress-related biomarkers in an atopic Dermatitis Model. Biomedicines 2021, 9(12). 10.3390/biomedicines9121764 PMC8698818 34944580

[37] Ruoslahti E (1981). Fibronectin. J Oral Pathol.

[38] Hubmacher D, Sabatier L, Annis DS, Mosher DF, Reinhardt DP (2011). Homocysteine modifies structural and functional properties of fibronectin and interferes with the fibronectin-fibrillin-1 interaction. Biochemistry.

[39] Bao H, Huo Q, Yuan Q, Xu C. Fibronectin 1: A Potential Biomarker for Ovarian Cancer. Dis Markers. 2021;5561651. 10.1155/2021/5561651 PMC8164534 34093898

[40] Zhang X, Liu H, Zhang J, Wang Z, Yang S, Liu D, Liu J, Li Y, Fu X, Zhang X (2023). Fibronectin-1: a predictive immunotherapy response biomarker for muscle–invasive bladder Cancer. Arch Esp Urol.

[41] Wu S, Liu C, Wei X, Nong WX, Lin LN, Li F, Xie XX, Liao XS, Luo B, Zhang QM (2022). High expression of fibronectin 1 predicts a poor prognosis in Glioblastoma. Curr Med Sci.

[42] Lenting PJ, Christophe OD, Denis CV (2015). Von Willebrand factor biosynthesis, secretion, and clearance: connecting the far ends. Blood.

[43] Lancellotti S, Sacco M, Basso M, De Cristofaro R (2019). Mechanochemistry of Von Willebrand factor. Biomol Concepts.

[44] Randi AM, Smith KE, Castaman G (2018). Von Willebrand factor regulation of blood vessel formation. Blood.

[45] Lykissas MG, Gelalis ID, Kostas-Agnantis IP, Vozonelos G, Korompilias AV (2012). The role of hypercoagulability in the development of osteonecrosis of the femoral head. Orthop Rev (Pavia).

[46] Zalavras C, Dailiana Z, Elisaf M, Bairaktari E, Vlachogiannopoulos P, Katsaraki A, Malizos KN (2000). Potential aetiological factors concerning the development of osteonecrosis of the femoral head. Eur J Clin Invest.

[47] Dal-Bo M, Del Giudice I, Bomben R, Capello D, Bertoni F, Forconi F, Laurenti L, Rossi D, Zucchetto A, Pozzato G (2011). B-cell receptor, clinical course and prognosis in chronic lymphocytic leukaemia: the growing saga of the IGHV3 subgroup gene usage. Br J Haematol.

[48] Matsuda M, Sugo T (2001). Hereditary disorders of fibrinogen. Ann N Y Acad Sci.

[49] Budzynski AZ (1986). Fibrinogen and fibrin: biochemistry and pathophysiology. Crit Rev Oncol Hematol.

[50] Shi J, Zeng S, Zhang Y, Zuo Z, Tan X (2023). Fibrinogen beta chain may be a potential predict biomarker for pre-eclampsia: a preliminary study. Clin Chim Acta.

[51] Repetto O, Maiero S, Magris R, Miolo G, Cozzi MR, Steffan A, Canzonieri V, Cannizzaro R, De Re V. Quantitative Proteomic Approach targeted to Fibrinogen beta chain in tissue gastric carcinoma. Int J Mol Sci. 2018; 19(3). 10.3390/ijms19030759 PMC5877620 29518939

[52] Wakabayashi I, Mambo N, Ueda T, Nonaka D, Lee LJ, Tanaka K, Kotani J (2018). New biomarkers for prediction of disseminated intravascular coagulation in patients with Sepsis. Clin Appl Thromb Hemost.

[53] Tung CL, Lin ST, Chou HC, Chen YW, Lin HC, Tung CL, Huang KJ, Chen YJ, Lee YR, Chan HL (2013). Proteomics-based identification of plasma biomarkers in oral squamous cell carcinoma. J Pharm Biomed Anal.

[54] Feng Z, Pan Y, Liu Y, Zhao J, Peng X, Lu G, Shi W, Zhang D, Cui S. Screening and analysis of serum protein biomarkers infected by Coronavirus Disease 2019 (COVID-19). Trop Med Infect Dis. 2022; 7(12). 10.3390/tropicalmed7120397 PMC9788497 36548652

[55] Sienko J, Teliga-Czajkowska J, Przytula E, Czajkowski K, Smolarczyk R, Nowis D (2019). Peroxiredoxin-1 as a prognostic factor in patients with ovarian cancer. Ann Agric Environ Med.

[56] Rhee SG, Woo HA (2011). Multiple functions of peroxiredoxins: peroxidases, sensors and regulators of the intracellular messenger H(2)O(2), and protein chaperones. Antioxid Redox Signal.

[57] Hopkins BL, Neumann CA (2019). Redoxins as gatekeepers of the transcriptional oxidative stress response. Redox Biol.

[58] Qiao Z, Xin M, Wang L, Li H, Wang C, Wang L, Tang T, Zhu B, Huang G, Wang Y (2020). Proteoglycan 4 predicts tribological properties of repaired cartilage tissue. Theranostics.

[59] Garusi E, Rossi S, Perris R (2012). Antithetic roles of proteoglycans in cancer. Cell Mol Life Sci.

[60] Thorson C, Galicia K, Burleson A, Bouchard O, Hoppensteadt D, Fareed J, Hopkinson W (2019). Matrix metalloproteinases and their inhibitors and Proteoglycan 4 in patients undergoing total joint arthroplasty. Clin Appl Thromb Hemost.

[61] Lee KY, Chuang HC, Chen TT, Liu WT, Su CL, Feng PH, Chiang LL, Bien MY, Ho SC (2015). Proteoglycan 4 is a diagnostic biomarker for COPD. Int J Chron Obstruct Pulmon Dis.

[62] Richendrfer H, Jay GD (2020). Lubricin as a therapeutic and potential biomarker in Sepsis. Crit Care Clin.

[63] Slovacek H, Khanna R, Poredos P, Poredos P, Jezovnik M, Hoppensteadt D, Fareed J, Hopkinson W (2021). Interrelationship of MMP-9, Proteoglycan-4, and inflammation in Osteoarthritis patients undergoing total hip arthroplasty. Clin Appl Thromb Hemost.

[64] Guo Y, Hu HT, Xu SJ, Xia WL, Zhao Y, Zhao XH, Zhu WB, Li FT, Li HL (2022). Proteoglycan-4 predicts good prognosis in patients with hepatocellular carcinoma receiving transcatheter arterial chemoembolization and inhibits cancer cell migration in vitro. Front Oncol.

[65] Martinez-Pinna R, Ramos-Mozo P, Madrigal-Matute J, Blanco-Colio LM, Lopez JA, Calvo E, Camafeita E, Lindholt JS, Meilhac O, Delbosc S (2011). Identification of peroxiredoxin-1 as a novel biomarker of abdominal aortic aneurysm. Arterioscler Thromb Vasc Biol.

[66] Cai CY, Zhai LL, Wu Y, Tang ZG (2015). Expression and clinical value of peroxiredoxin-1 in patients with pancreatic cancer. Eur J Surg Oncol.

[67] Zhang Y, Wu XX, Li S, Wu JF, Han S, Lin ZJ, Ding SZ, Gong WJ (2020). Peroxiredoxin 1 as an inflammatory marker in diarrhea-predominant and postinfectious irritable bowel syndrome. Neurogastroenterol Motil.

[68] Verp MS, Amarose AP (1987). Inflammatory bowel disease and X chromosome abnormalities. A case report. J Reprod Med.

[69] Aida I, Ozawa T, Fujinaka H, Goto K, Ohta K, Nakajima T (2021). Autosomal recessive spastic Ataxia of Charlevoix-Saguenay without Spasticity. Intern Med.

[70] Liu X, Wang C, Meng H, Liao S, Zhang J, Guan Y, Tian H, Peng J (2022). Research Progress on exosomes in Osteonecrosis of the femoral head. Orthop Surg.

[71] Li L, Ding Y, Liu B, Wang Z, Carlone DL, Yu X, Wei X, Zhang F, Lineaweaver WC, Yang B (2021). Transcriptome landscape of the late-stage alcohol-induced osteonecrosis of the human femoral head. Bone.

[72] Guo M, Zhang J (2023). Metabolomic analysis of bone-derived exosomes in osteonecrosis of the femoral head based on UPLC-MS/MS. Metabolomics.

